# Indentity of Typhus and Typhoid Fever

**Published:** 1847-04

**Authors:** 


### ECLECTIC DEPARTMENT,

### AND SPIRIT OF THE MEDICAL PERIODICAL PRESS.

---

*Indentity of Typhus and Typhoid Fever*.—The question whether the forms of fever called Typhus and Typhoid are identical or essentially distinct, has excited, of late, considerable discussion; Drs. Gerhard, Bartlett and others, contending that they are in fact, two diseases, while the majority of English and American writers, regard them as varieties of the same disease. In view of the pathological interest of the subject, and, the importance of bringing it before the attention of the profession, in order that the question may be settled by a more extensive collection of facts, we present to our readers a chapter devoted to it, by Prof. Clymer, in the work on Fevers lately published under his editorship, of which we have made mention in previous Numbers of this Journal. Prof. C., it will be perceived, is disposed to think that the evidence of their non-identity is not conclusive.—ED. BUFFALO MED. JOUR.

"The question of the identity of typhus and typhoid fever has been one of great interest, and respecting which much diversity of sentiment at one time prevailed. Whilst the British physicians held, with great unanimity, the opinion that, though different varieties, they were the same species of fever; the French and American authorities proclaimed their essentially dissimilar nature. For obvious reasons the present work is not suitable for the discussion of this point; and it is our intention merely to state the grounds on which those who contend for the non-identity of the two diseases base their opinions, to ascertain how far they are in accordance with the observed facts. The leading features of difference insisted on to prove that the two varieties of fever—typhus and typhoid—are radically distinct, are, 1. The character of the eruption, which in typhoid fever consists of minute lenticular papulæ, disappearing on pressure, and thinly scattered over the abdomen and chest; whilst in typhus the spots "are more irregular in their shape and size; not elevated above the adjacent skin; partially disappearing on pressure, or not at all; often abundant, or even confluent; in many cases occupying the skin of the extremities as well as that of the entire trunk, and usually of a duller and more dusky color than in the former disease."* 2. The infrequency of the abdominal symptoms—diarrhœa, and pain and gurgling on pressure in the right iliac region—in typhus, which occurs so constantly in typhoid fever; and 3. The absence in typhus of the characteristic intestinal lesions—specific alteration of the agminate and isolated follicles—of typhoid fever.

"Let us briefly examine how far these asserted differences are sustained by observation, assuming, for the purpose of having some distinctive or pathognomonic trait assigned to typhoid fever, that a diseased condition of the glands of Peyer is necessary to constitute the disease.

"Dr. Gaultier de Claabry† has demonstrated the complete identity of the camp and jail fevers of the Continent of Europe, with the typhoid fever of Paris, which, again, has been proved to be identical with the prevalent form of continued fever in the northern and middle sections of this country. The typhus of camps, hospitals, and prisons, which prevailed so constantly in every part of Europe, at different periods, from 1742 to 1815, was, as to symptomatology, march and pathology, the form of fever described by

* BARTLETT, loc. cit., p. 270.
† De l'Identite du Typhus et de la Fievre Typhoide, &c, &c., Paris, 1844.

Louis, Chomel, Forget, &c. In the last edition of his work on Typhoid Fever (1841) Dr. Louis has recognized the perfect identity of two diseases.

"Between the first of October, 1839, and April, 1840, an epidemic continued fever prevailed in the prison at Rheims, in France, and which has been faithfully described by Dr. Landouzy.* The epidemic in question was confined to the inmates of a certain quarter of the prison at Rheims, and prevailed between the first of October, 1839, and April, 1840. The entire number of cases was 138, 103 of which were amongst the inmates of the prison; the remaining 35 consisting of physicians, medical students, nurses, and others connected with the hospital where the patients were treated. An important point connected with these cases is, that they all came under the observation of the medical attendants immediately on the commencement of the disease. Among the first symptoms was stupor, which frequently showed itself as early as the second or third day, and continued until it was lost in coma or delirium. It differed from mere somnolence and coma. The expression of the countenance was that of half-demented and stupid astonishment.—It was the *stupor attonitus* of Foes. In half the cases it was strongly marked, in the other half it was slight in degree. True somnolence and coma appeared in a certain number of cases, later in the disease, often about the tenth day. Profound coma, so that the patient could not be roused, existed in only twelve cases. Delirium was very common, usually making its appearance between the third and the eighth day. It was generally low and muttering in its character, and, in fatal cases, it continued until death. Headache was uniformly present at the commencement of the disease.—It was, for the most part, dull and heavy, and felt especially over the eyes. It continued for an uncertain period of time, gradually disappearing or losing itself in coma or delirium. *Subsultus tendinum* was common, and strongly marked in grave cases. Redness of the eyes, *tinnitus aurium*, and deafness were present in a certain proportion of cases, but differed in no obvious particulars from the same symptoms in typhoid fevers. There was great loss of muscular strength, from the beginning of the disease. In every case except the first, which was not carefully examined, there was an abundant cutaneous eruption, consisting of small spots, or *ecchymoses*, of a red, violet, or black colour, not elevated above the skin, and not disappearing on pressure. They were always found on the chest, often also on the abdomen, and in some cases they extended to the arms and legs. They commonly showed themselves about the fourth or fifth day, and gradually faded away between the tenth and the eighteenth. They were abundant and confluent in proportion to the gravity of the disease. The bodies of the sick exhaled a strong, offensive odor, resembling that of mice. In regard to the absence

* Archives Generales de Medecine, &c., Paris, 1842. BARTLETT, loc. cit., p. 302.

of appetite, to thirst, the state of the lips, tongue and mouth, nothing special was observed, differing from what occurs in typhoid fever. Nausea was present at the commencement of the disease in all the cases. *Meteorism and abdominal pains were uniformly absent.*— There was diarrhœa in the beginning of the disease in only four cases. In all the others there was no apparent disturbance in the functions of the intestinal canal. The bowels were more inclined to constipation than to looseness. A distinct, well-marked sibilant rhonchus was present in all the cases. Epistaxis occurred in eight cases. The temperature of the surface was uniformly elevated ; the heat was dry and burning. In no instance was there gangrene of any part of the body. In the six autopsies which were performed the intestinal lesions characteristic of typhoid fever were present. The elliptical plates were either thickened and elevated, or they were the seats of ulcerations ; and the mesenteric glands corresponding to them were enlarged. *The spleen was not increased in size in any of the cases :* in four it seemed somewhat softened.

On the 17th of January, 1842, an epidemic fever broke out in the barrack of the municipal guard at Stockholm. The corps had been remarkably healthy for the five previous years, and occupied its usual quarters, which were very damp, especially in winter. The men were lodged in large and spacious wards, fourteen feet high, in each of which forty-four persons slept, two in a bed. The food and clothing were of the best description. For two months previous to the occurrence of the epidemic, the men were in the habit of returning to their quarters almost drenched to the skin, and their wet clothes were hung up in the room in which they slept. The atmosphere was loaded with moisture, and fresh air was admitted as sparingly as possible, in order that an elevated temperature might be maintained. The age of the patients ranged from 19 to 52 :— 23 were under 25 years ; 20 from 25 to 30 ; 12 from 30 to 35 ; 6 from 35 to 40 ; and 3 from 40 to 53. Of 64 persons attacked 62 recovered, The cases are divided into three categories. A. Cases with predominance of cerebral symptoms, (*twenty-two cases.*) B. Cases with predominance of abdominal symptoms, (*twelve cases.*)— C. Mixed cases, (*thirty cases.*) The premonitory symptoms, which were seldom entirely wanting, were prostration, anorexia, dizziness, pains in the head and loins, with occasional chills and troubled sleep, during an average period of three days. In classes A and C epistaxis now and then occurred ; and in B and C diarrhœa, nausea, or colic, was sometimes met with during the period of incubation.— The subsequent symptoms were those of typhoid fever. In classes B and C there were, in nearly all, ileo-cæcal pain and gurgling on pressure, with diarrhœa ; whilst in A there was constipation, and the belly was soft and free from pain ; in all the cases there was an eruption, consisting of reddish spots, of irregular form, and varying in magnitude, from a minute dot to the size of a bean, disappearing momentarily on pressure, more or less abundant on the chest and shoulders, very rarely seen on the face or abdomen, bright red at first, usually on the third or fourth day they gradually became darker, assuming frequently a bluish or violet-blue tint. The duration of an attack was about fifteen days. Of the two deaths, one took place on the fifth day, when the heart was found to be softened, the blood decomposed, the spleen broken down, (*enbouille*,) and Peyer's glands enlarged, but not ulcerated. In the other fatal case convalescence had commenced on the thirteenth day, when the patient undergoing some exposure, pneumonia occurred, and he died on the seventeenth day. The spleen was softened, with a bluish-gray colour of Peyer's glands, "with some of the orifices of their ducts evidently enlarged."*

Dr. Felix Jacquot, a French Army Surgeon, had opportunities of observing epidemics of typhoid fever at Paris, Lyons, Vosges, Strasburg, and Metz. At Lyons, the lenticular spots, with petechiæ and ecchymoses, were frequent. At Metz, sudamina were occasionally met with, and nothing else eruptive was seen. At Lyons, pulmonic complications were rare, whilst at the civic hospitals they were so common, that Dr. Dupasquier was led to believe that pneumonia was the anatomical character of the disease. In one epidemic, diarrhœa was a prominent, early and constant symptom; whilst in another constipation was general.†

Dr. John P. Mettaurer, in an excellent paper on Continued Fever, as it prevailed in Middle Southern Virginia, from 1816 to 1829 inclusive—a period of thirteen years‡—says, "these varieties of fever have prevailed at different times; that is, the Synocha, the Typhoid, and the Typhus. Synocha, which was only the more open and well-developed form of the disease, prevailed during dry and warm, and warm and damp seasons, and always as an endemico-epidemic of considerable extent. From 1816 to 1821, both years included, and especially during the warm months of those years, this was the predominant form under which continued fever appeared; and it was uniformly distinguished by the open characters of a well-marked inflammatory fever in a large majority of cases, throughout its more acute stage. During these years, too, all classes and conditions of society were equally affected by the disease, and, with few exceptions, it did not part with its peculiar open characters until the setting in of cold weather. Many cases of ordinary continued fever, or as it is often termed typhoid fever, and typhus, also occurred during this period, though their number was small until after winter set in. From 1822 to 1829, the typhoid and typhus varieties were the predominant fevers, but there were also many cases of the synocha type to be met with, both during the warm and cold seasons of this period; and these fevers were almost exclusively confined to certain districts and neighborhoods,

* Gaz. Med., t. xii., Nos, 15 to 26, 1845.

† Gazette Medicale, t. xii., Nos. 33, 34, Paris, 1845.

‡ Am. Journ. Med. Sciences, July, 1843.

favourable to the generation of a peculiar miasm, such as is believed to be formed during the slow decomposition of ligneous substances. Typhus did not generally make its appearance until many cases of the typhoid affection had previously occurred in a family; but when it did take place, it was invariably distinguished by early cerebral disturbances, especially depression of the nervous energies. The face of the country and climate of the region in which this fever prevailed, presented nothing calculated to lead to the belief that it was necessarily obnoxious to the disease. The lands are more or less broken and undulating; they abound in numerous small water-courses, and creek-flats which readily overflow; and many of the small streams dry up during protracted droughts. The climate is not more variable than is usual in middle Virginia. With the exception of two of the years embraced in this history, there was nothing very remarkable or peculiar in the seasons favouring the production of continued fever. 1816 was distinguished by an unusually dry and cool summer. From May until October, there was not rain enough to wet the earth an inch, consequently nearly all the water-courses large and small, dried up. There were many instances of wells and springs failing entirely during this year, which had never been known to be materially affected by droughts before. And the summer of this extraordinary season was so cool as to be attended with frost every month. This was a year of fever, and the disease assumed the synocha form, distinguished by well developed, and highly inflammatory characters; and it prevailed most extensively, especially during the warm months. Towards the close of September, the cases of typhoid fever, which until then had not been numerous, multiplied rapidly, and soon became decidedly the predominant form, and maintained the ascendency during the succeeding winter. In 1827, another exceedingly dry summer occurred: this year was also distinguished by the almost universal prevalence of the synocha form of continued fever, though there were occasional cases of the typhoid and typhus varieties likewise to be met with. The summer of this year was very nearly as dry as that of 1816, but it was exceedingly warm. As autumn approached, typhoid cases increased in number, and soon became the predominant fevers. But after winter set in, which was damp and remarkably mild, genuine typhus rapidly increased, and continued to do so the remainder of he season, and throughout the next year. The years not particularized in the foregoing sketch, were not distinguished by anything remarkable in a meteorological view, but they were years of fever; and the disease appeared chiefly under the typhoid and typhus forms, nearly in an equal proportion, though cases of synocha were often met with likewise. 1816 and 1817 were decidedly the sickly years of the period embraced in this paper; and, as already remarked, the synocha was the predominant form under which the disease appeared. In many instances the fevers of these years could be satisfactorily traced to malarial sources, especially at the commencement of the sickly season. But a vast number afterwards were as clearly referable to idiomiasma, or something like contagion, particularly as cases of fever multiplied in families, and as cold weather approached, or as the rigors of winter increased. Numerous cases occurred after the cold weather of the winter season set in, with individuals residing in uninfected neighborhoods, who had assisted in nursing sick relatives ; and such persons often communicated the disease to their famalies and neighbors who had assisted in nursing them. The propagation of every form of this fever by something like contagion was established by indubitable evidence in many instances ; but it was not very communicable until it had continued in a family some time, more especially the synocha and typhoid varieties. The difference in the communicability of these forms and typhus was, that many cases of the former, or one of long continuance became necessary to their propagation by the contagious agency ; while a solitary example of the latter in many instances was sufficient to communicate it. In a few instances we were induced to believe that fomites propagated these fevers, as washerwomen residing in neighborhoods remote from the seats of the disease, who had received and washed the clothes of the sick, and who were exposed in no other manner, had regular attacks of them.

"'No age was exempted from continued fever ; infants at the breast, and individuals upwards of sixty years old, with every intermediate age, being affected. Being between the ages of eighteen and thirty years was that most obnoxious to this fever. The black population were most subject to typhus, and the depressing forms of the typhoid variety.

"'The disease was frequently superinduced by catarrhal affections, especially during variable seasons. Measels ; hooping-cough; chicken-pox ; worms ; conception ; parturition ; excesses in eating and drinking ; surgical accidents and operations ; terror ; passion ; grief; despondency ; intense study ; long fasting; colic ; fatigue ; and some others, at different times, also seemed to excite the disease, but as accidental or occasional causes.

"'The *anatomical characters* which were displayed during our post-mortem examinations* were lesions of the glands of Peyer ; of the mesentery ; spleen ; mucous membrane of the small and large intestines ; of the peritoneal coat of the small intestines and stomach ; of the meninges of the brain, and especially of the arachnoid ; effusion of serum, or blood and serum into the ventricles of the brain ; congestion or softening of the lungs, and gangrene of the bladder. A dissolved state of the blood was also occasionally met with as a post-obit appearance, especially in fatal cases of typhus : in such cases the lungs were more or less disorganized ; and in every instance the membranes of the brain were greatly congested.'

* "We treated more than four hundred cases from 1816 to 1829, and lost only twenty patients. Many of the cases were of the most unpromising description, perhaps a fifth."

"An epidemic of continued fever which occurred at the Lane Seminary, Ohio, during the autumn of 1842, and the ensuing winter, has been described by Dr. Thomas Carroll, of Cincinnati, in the Western Journal of Medicine and Surgery, for 1843. In nearly all the cases diarrhœa, meteorism, and pain and gurgling in the ileo-cœcal region, are mentioned as present, and yet in many of the fatal cases Peyer's glands were perfectly healthy; but ulcerations were frequent in the large intestines.

In October, 1845, the writer saw several cases of continued fever in the wards of the Philadelphia Hospital, in emigrants just arrived, in which there existed, along with the abdominal symptoms of typhoid fever,—diarrhœa, and pain and gurgling on pressure in the right iliac region—the maculated eruption he had previously seen in the typhus of Dublin and London.

From these facts—few, it must be acknowledged, yet positive as far as they go—it is evident, 1. That there is no necessary or constant connection between the kind of eruption, and the abdominal symptoms or lesions;—that the lenticular eruption of the Paris fever, and the maculated, measly eruption of the Irish typhus may exist conjointly with diarrhœa, and pain and gurgling in the ileo-cœcal region, and with enlarged or ulcerated patches of Peyer. And 2. That there is no constant relation between the abdominal symptoms, pain, gurgling, and diarrhœa—and the intestinal lesions, since all these symptoms may be absent, not only in individual cases, but in a portion, or in the whole of those attacked in the course of a given epidemic.

"We would enquire whether the group of symptoms known as typhoid fever is perfectly indentical at all times and at all places; and further, if the essential characters of typhus are so constant or defined, that it can be taken as a fixed term of comparison? Do not epidemics of typhoid fever differ in their prominent phenomena, constantly, from sporadic cases, as well as from each other? Are there not a number of accessory circumstances, so subtile as to escape observation, perpetually modifying the influence of causes on the organism? General conclusions should not be drawn from limited observations, or from witnessing any disease so generally prevalent as the one under consideration, in a single district, or even country, and during a limited period of time. And we cannot avoid thinking that those who regard these two forms of fever as distinct and separate *species*, are premature in the expression of an opinion upon a subject in which there are, as yet, wanting many essential elements to warrant a positive judgment."

